# Avian Influenza A Virus Polymerase Can Utilize Human ANP32 Proteins To Support cRNA but Not vRNA Synthesis

**DOI:** 10.1128/mbio.03399-22

**Published:** 2023-01-16

**Authors:** Olivia C. Swann, Amalie B. Rasmussen, Thomas P. Peacock, Carol M. Sheppard, Wendy S. Barclay

**Affiliations:** a Department of Infectious Diseases, Faculty of Medicine, Imperial College London, London, United Kingdom; Boston University School of Medicine

**Keywords:** ANP32A, ANP32B, RNA polymerases, avian viruses, influenza, viral replication, virus-host interactions, zoonotic infections

## Abstract

Host restriction limits the emergence of novel pandemic strains from the influenza A virus avian reservoir. For efficient replication in mammalian cells, the avian influenza RNA-dependent RNA polymerase must adapt to use human orthologues of the host factor ANP32, which lack a 33-amino-acid insertion relative to avian ANP32A. Here, we find that influenza polymerase requires ANP32 proteins to support both steps of genome replication: cRNA and vRNA synthesis. However, avian strains are only restricted in vRNA synthesis in human cells. Therefore, avian influenza polymerase can use human ANP32 orthologues to support cRNA synthesis, without acquiring mammalian adaptations. This implies a fundamental difference in the mechanism by which ANP32 proteins support cRNA versus vRNA synthesis.

## INTRODUCTION

Influenza A viruses (IAVs) pose a pandemic risk: while the natural hosts of IAV are aquatic birds, the virus is associated with sporadic zoonotic jumps, which may trigger widespread disease in an immunologically naive population (1). The enormous consequences of such events are illustrated by the historical 1918 “Spanish flu” pandemic, as well as the coronavirus pandemic caused by SARS-CoV-2.

Prior to emerging as a pandemic strain, zoonotic IAV must adapt to overcome multiple host range barriers. One important restriction is adaptation of the RNA-dependent RNA polymerase (FluPol) for efficient activity within the mammalian cellular environment (1, 2). FluPol is a heterotrimer consisting of three viral proteins: PB1, PB2, and PA. Each of the eight viral genomic RNAs (vRNAs) of the IAV genome are encapsidated by nucleoproteins (NPs) and associate with a FluPol protomer in a viral ribonucleoprotein complex (vRNP). During infection, FluPol drives both transcription and replication of the viral genome (3). Transcription occurs in a primer-dependent manner to produce capped and polyadenylated positive sense viral mRNAs. Genome replication occurs in a two-step process: first, negative sense vRNA is copied into a full-length positive-sense cRNA intermediate which is packaged into complementary RNPs (cRNPs) by acquiring an encapsidating polymerase and NP. Nascent vRNPs are then produced from cRNPs (3, 4). Although both cRNA and vRNA synthesis undergo *de novo* initiation without any primer, the mechanism differs. While cRNA synthesis is initiated at the terminus, vRNA synthesis uses internal initiation followed by primer realignment that is dependent on an additional transactivating FluPol (5–8).

Structural studies show how the functional flexibility of FluPol is possible due to rearrangements of peripheral domains outside the catalytic core (3). These depend on the presence or absence and nature of the bound RNA promoter, or upon dimerization with additional polymerase molecules (9–11). FluPol also coopts host factors to coordinate its activity, some of which may stabilize different functional states of the polymerase (10–13).

ANP32 proteins are a family of proviral host factors that are essential for influenza replication (14, 15). Species differences between ANP32 proteins underlie host restriction of avian influenza polymerase in mammalian cells (16). Prototypical avian influenza polymerase bearing a glutamic acid at position 627 of the PB2 protein (here referred to as FluPol 627E) cannot be supported by mammalian ANP32 proteins that lack a 33-amino-acid insertion present in avian ANP32A orthologues and, accordingly, show restricted replication in human cells. In contrast, FluPol bearing a single amino acid substitution at the PB2 627 position from glutamic acid to lysine (the quintessential mammalian-adapting mutation, FluPol 627K), can utilize mammalian ANP32 proteins that lack this insertion, and such viruses replicate to high titers in human cells (2, 16). Interestingly, human ANP32A binds FluPol 627E, despite not supporting function (17–19).

Earlier studies suggest that ANP32 proteins are specifically required to support vRNA synthesis (20), and that host restriction occurs at this step of replication (21, 22). Nevertheless, more recent reports implicate ANP32 in both steps of replication (11, 23). The recent structures of huANP32A and chANP32A in complex with FluPol from influenza C virus and a short 47-nucleotide vRNA demonstrate that the N-terminal leucine rich repeat (LRR) domain of ANP32 bridges a novel asymmetric dimer of two FluPol heterotrimers. This dimer was interpreted as a “replication complex,” with the RNA-bound FluPol (FluPol_R_) adopting a replication-competent structure, and the additional apo-enzyme (FluPol_E_) in a novel conformation poised to encapsidate the nascent RNA into an RNP complex. Interestingly, mutations introduced to disrupt the FluPol_E_-FluPol_R_ interface resulted in a significant reduction in cRNA accumulation. This, combined with the logic that both cRNA and vRNA require encapsidation into RNPs during replication, suggests that ANP32 may play a role in cRNA synthesis, as well as in vRNA synthesis (11). However, if this is the case, then it is not clear why host range restriction would occur only at vRNA synthesis, and not at the first, pioneering round of cRNP formation.

Here, we use ANP32 knockout cell lines to clarify the role of ANP32 proteins in IAV cRNA synthesis. We establish an RNA fluorescence *in situ* hybridization (FISH) assay for directly visualizing cRNA and demonstrate that ANP32 proteins are essential for primary cRNA synthesis in authentic infection, as well as under experimental conditions in which vRNA synthesis is inhibited. Nevertheless, we find that avian FluPol 627E does not show restricted cRNA accumulation in mammalian cells. This observation is consistent regardless of whether FluPol_R_, FluPol_E_, or both FluPol molecules in the replication complex bear the avian-like 627E signature. Moreover, the cRNPs produced by FluPol_R_ 627E are functional for onward replication. To conclude, our study suggests that host restriction of avian influenza polymerase in human cells acts specifically at the level of vRNA synthesis.

## RESULTS

### cRNA synthesis is inhibited in human cells lacking ANP32A/B.

**FIG 1:**
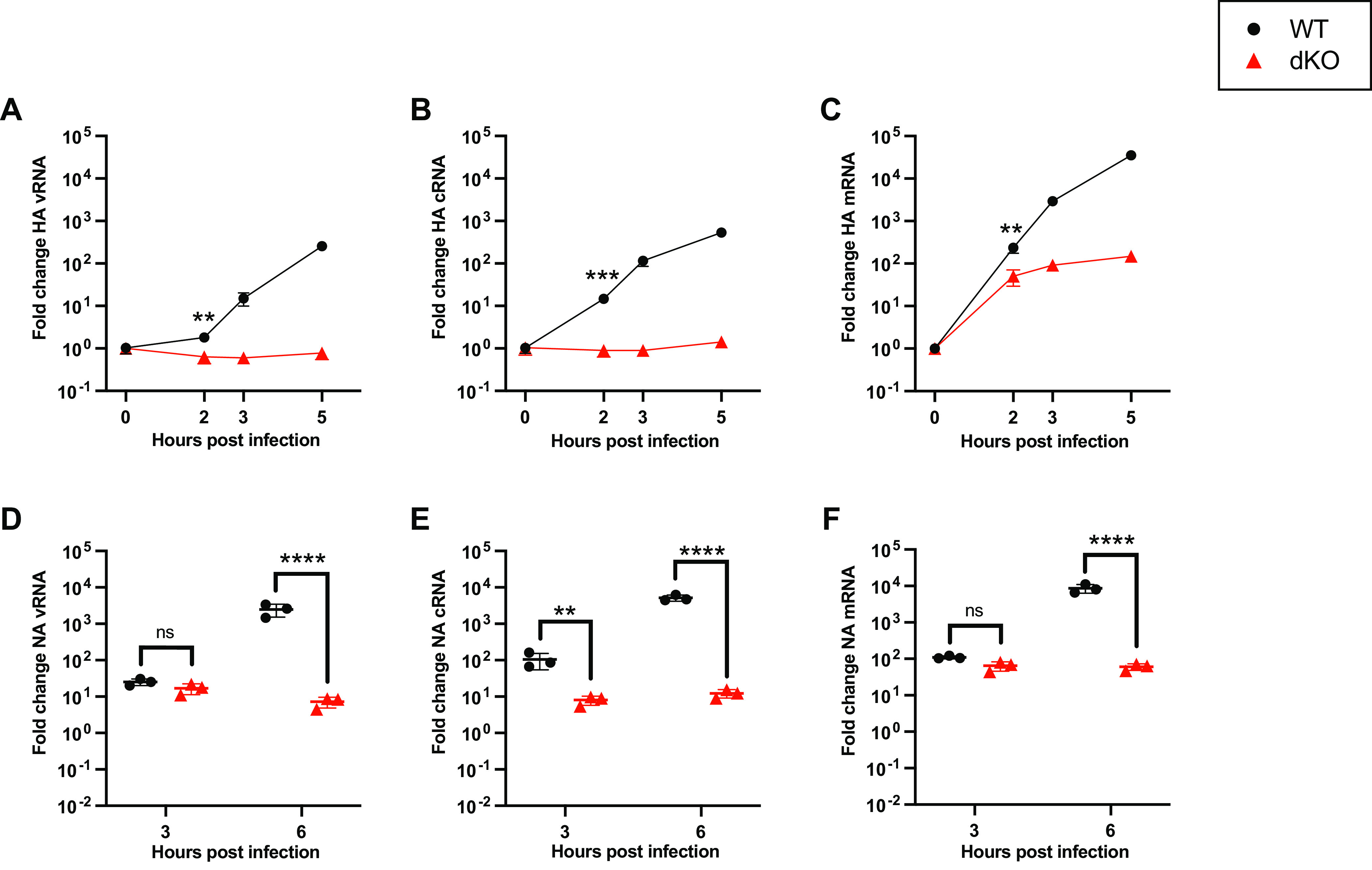
cRNA synthesis is inhibited in human cells lacking ANP32A/B. (A to C) Segment 4, vRNA (A), cRNA (B), or mRNA (C) accumulation over time in eHAP WT versus dKO cells following infection with PR8 (MOI = 3). The fold change was calculated versus input (0 hpi). *n* = 3 biological replicates, plotted as means ± the standard deviations (SD). Significance between WT and dKO cells was assessed at 2 hpi using an unpaired *t* test following log transformation. (D to F) Segment 6 vRNA (D), cRNA (E), or mRNA (F) accumulation over time in eHAP WT versus dKO cells after infection with PR8 (MOI = 3). The fold change was calculated versus mock-infected cells. *n* = 3 biological replicates, plotted as means ± the SD. Significance was assessed using multiple unpaired *t* tests following log transformation and corrected for multiple comparisons using the false discovery rate. ns, not significant; **, *P* < 0.01; ***, *P* < 0.001; ****, *P* < 0.0001.

Human cells express three members of the ANP32 protein family: huANP32A, huANP32B, and huANP32E (24). Previous work has demonstrated that huANP32A/B are functionally redundant to one another in their proviral activity, while huANP32E does not support IAV FluPol activity (14, 15). To investigate the role of ANP32 proteins in cRNA synthesis, wild-type human eHAP cells (eHAP WT) and human eHAP cells in which ANP32A/B have been ablated (eHAP dKO) (14) were infected with A/Puerto Rico/8/1934(H1N1) (PR8), and the accumulation of segment 4 (hemagglutinin [HA]) vRNA, cRNA, and mRNA was quantified using a tagged reverse transcription-quantitative PCR (RT-qPCR) approach (25). To minimize the indirect effect of reduced vRNP templates on cRNA synthesis, we focused on early time points of infection. Following infection, there was no increase in either vRNA or cRNA levels over time in the absence of ANP32A/B, confirming ANP32 proteins are essential for replication (Fig. 1A and B). Significantly, no cRNA accumulation was observed over input, suggesting a direct role for ANP32 proteins in the pioneering round of cRNA synthesis. In contrast, and as expected, HA mRNA transcripts increased 50-fold in dKO cells from 0 h postinfection (hpi) to 2 hpi, since primary transcription does not require ANP32 proteins (Fig. 1C). In separate experiments, we analyzed the accumulation of segment 6 (neuraminidase [NA]) vRNA, cRNA, and mRNA in eHAP WT and dKO cells, using an analogous tagged RT-qPCR approach. As with segment 4, at later time points, a significant reduction in all three RNA species was observed (Fig. 1D to F). At 3 hpi, an ~10-fold reduction in the accumulation of cRNA was already apparent in the dKO cells, despite no difference in vRNA accumulation having yet occurred. Again, this suggests a direct role for huANP32A/B in supporting cRNA synthesis.

### cRNA can be visualized using *in situ* replication assays.

**FIG 2:**
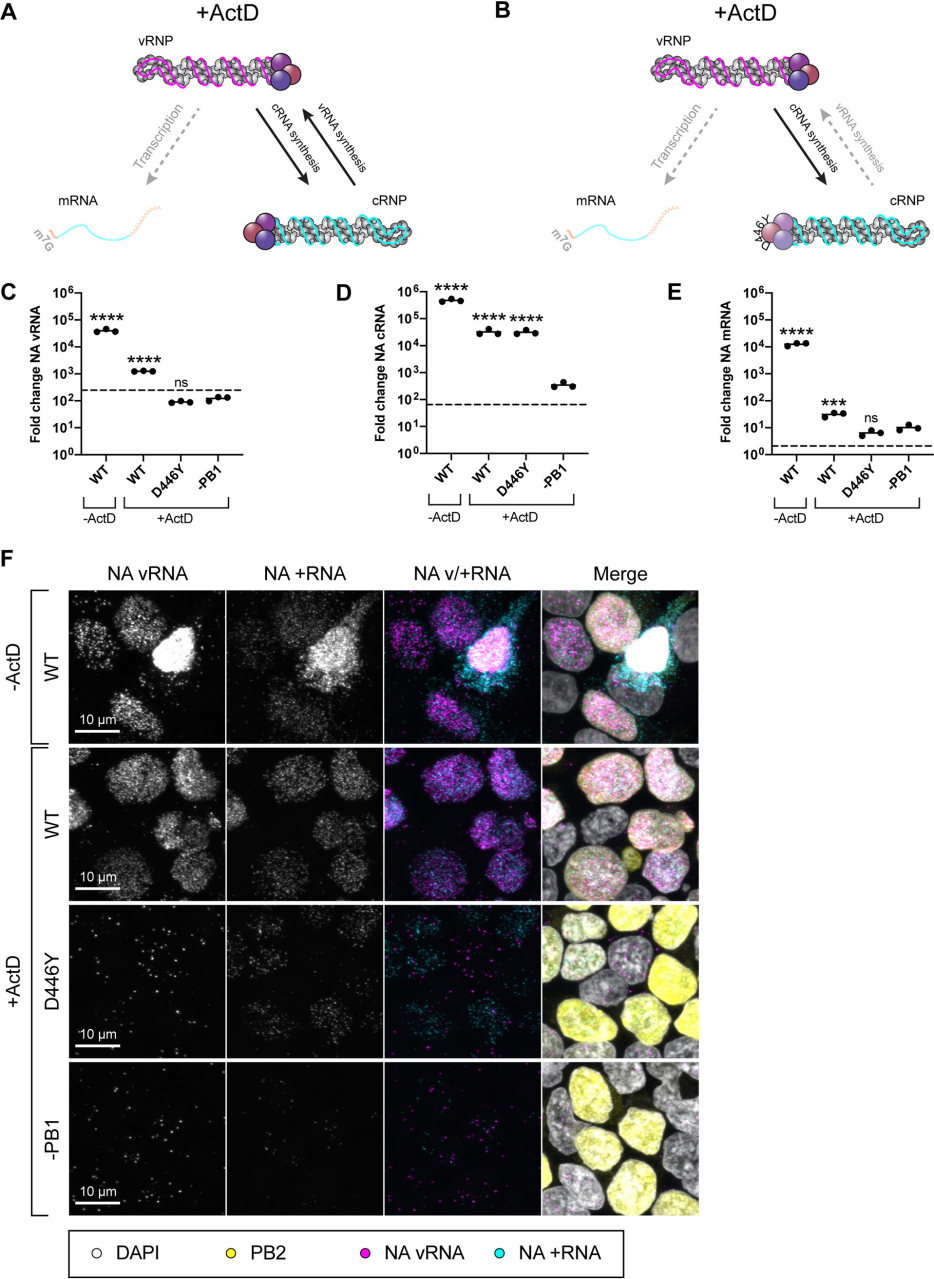
cRNA can be visualized using *in situ* replication assays. (A) Schematic illustrating influenza polymerase activity under the conditions of an ActD-treated replication assay. (B) Schematic illustrating influenza polymerase activity under the conditions of an ActD-treated cRNP stabilization assay. (C to F) Validation of ActD-treated replication and cRNP stabilization assays analyzed by RNA FISH. Assays were performed in HEK293T cells, following infection with PR8 (MOI = 3). Pretransfected PR8 polymerase mixes were: WT = PB2, PB1, PA, and NP (replication assay); D446Y = PB2, PB1 D446Y, PA, and NP (cRNP stabilization assay); −PB1 = PB2, PA, and NP (control). (C to E) RT-qPCR analysis of segment 6 vRNA (C), cRNA (D), and mRNA (E) accumulation at 6 hpi. Pretransfected polymerase mixes are indicated on the *x* axis. The dotted line indicates input levels of RNA (0 hpi). Fold change was calculated versus mock-infected cells. *n* = 3 biological replicates, plotted as means ± the SD. Significance compared to −PB1 was assessed using one-way ANOVA with Dunnett’s multiple-comparison test, following log transformation. (F) Accumulation of NA vRNA and +RNA at 3 hpi, analyzed by RNAscope with indirect immunofluorescence against PB2. Pretransfected PR8 polymerase mixes are indicated on the left-hand side. Images are representative maximum-intensity projections. ns, not significant; ***, *P* < 0.001; ****, *P* < 0.0001.

To further investigate the role of ANP32 in FluPol replication, we developed an *in situ* assay for direct visualization of FluPol replication products that allows single cell, spatial information to be collected alongside bulk assay readouts. To achieve this, the RNA FISH assay RNAscope was used (26). Probes were designed to target either segment 6 negative sense RNA (NA vRNA probe) or positive sense RNA (NA +RNA probe). In a standard infection, the NA +RNA probe is unable to distinguish NA cRNA/mRNA due to the minimal sequence differences between these two RNA species. To counter this, we made use of established assays in which cellular conditions are manipulated to specifically inhibit different aspects of FluPol activity (21, 22, 27). Precisely, we made use of actinomycin D (ActD)-treated replication assays or cRNP stabilization assays, in which only replication (Fig. 2A) or only the primary round of cRNA synthesis (Fig. 2B) can occur, respectively. Viral transcription is inhibited in both assays. Thus, positive sense staining in these assays can be attributed specifically to cRNA. In brief, both assays first require exogenous expression of RNP components (FluPol/NP) that allow encapsidation of nascent cRNA/vRNA in the absence of viral transcription. For a replication assay WT FluPol is expressed, while for a cRNP stabilization assay a catalytically dead mutant (PB1 D446Y) is expressed that allows stabilization but not onwards replication. Subsequently, cells are treated with the transcriptional inhibitor ActD during infection, which indirectly inhibits viral transcription.

Characterization of this approach using RT-qPCR at 6 hpi (Fig. 2C to E) confirmed that vRNA and cRNA significantly accumulate over background (controls lacking transfected PB1 [-PB1]) during a replication assay (WT), only cRNA significantly accumulates during a cRNP stabilization assay (D446Y), and ActD treatment inhibits mRNA accumulation to <0.2% untreated levels (greater than 550-fold reduction). In the absence of ActD (−ActD, WT), *in situ* analysis revealed significant accumulation of vRNA and +RNA in the nuclei and cytoplasm of infected cells (Fig. 2F). Similarly, in a replication assay (+ActD, WT), strong NA vRNA/+RNA staining was detected. Crucially, however, this staining exclusively localized to the nucleus. This implies efficient inhibition of transcription, as we expect export of NA transcripts by this time point, as seen in the untreated control. Moreover, vRNP export is dependent on the expression of additional viral proteins that are not pre-expressed in this assay. Thus, a lack of vRNP export implies expression of these proteins from the incoming virus is successfully inhibited. In a cRNP stabilization assay (+ActD, D446Y), +RNA accumulation was observed throughout the nuclei of transfected cells; however, only sparse punctate NA vRNA staining was detected. This likely corresponds to input genomes as a similar level of NA vRNA staining was visible under conditions in which nascent vRNPs cannot be stabilized (+ActD, −PB1). The minimal NA +RNA staining detected in this control indicates the background level of viral transcription in the presence of ActD, plus any nonspecific staining, and confirms that the majority of +RNA staining in the replication and cRNP stabilization assays can be attributed to cRNA. No difference in transfection efficiency was observed, either by anti-PB2 staining, or by Western blot against NP (see Fig. S1 in the supplemental material).

10.1128/mbio.03399-22.1 FIG S1 Matched Western blot for Fig. 2C to F. Download FIG S1, PDF file, 0.1 MB. Copyright © 2023 Swann et al. 2023 Swann et al. https://creativecommons.org/licenses/by/4.0/ This content is distributed under the terms of the Creative Commons Attribution 4.0 International license.

### Human ANP32 proteins play a direct role in primary cRNA synthesis.

**FIG 3:**
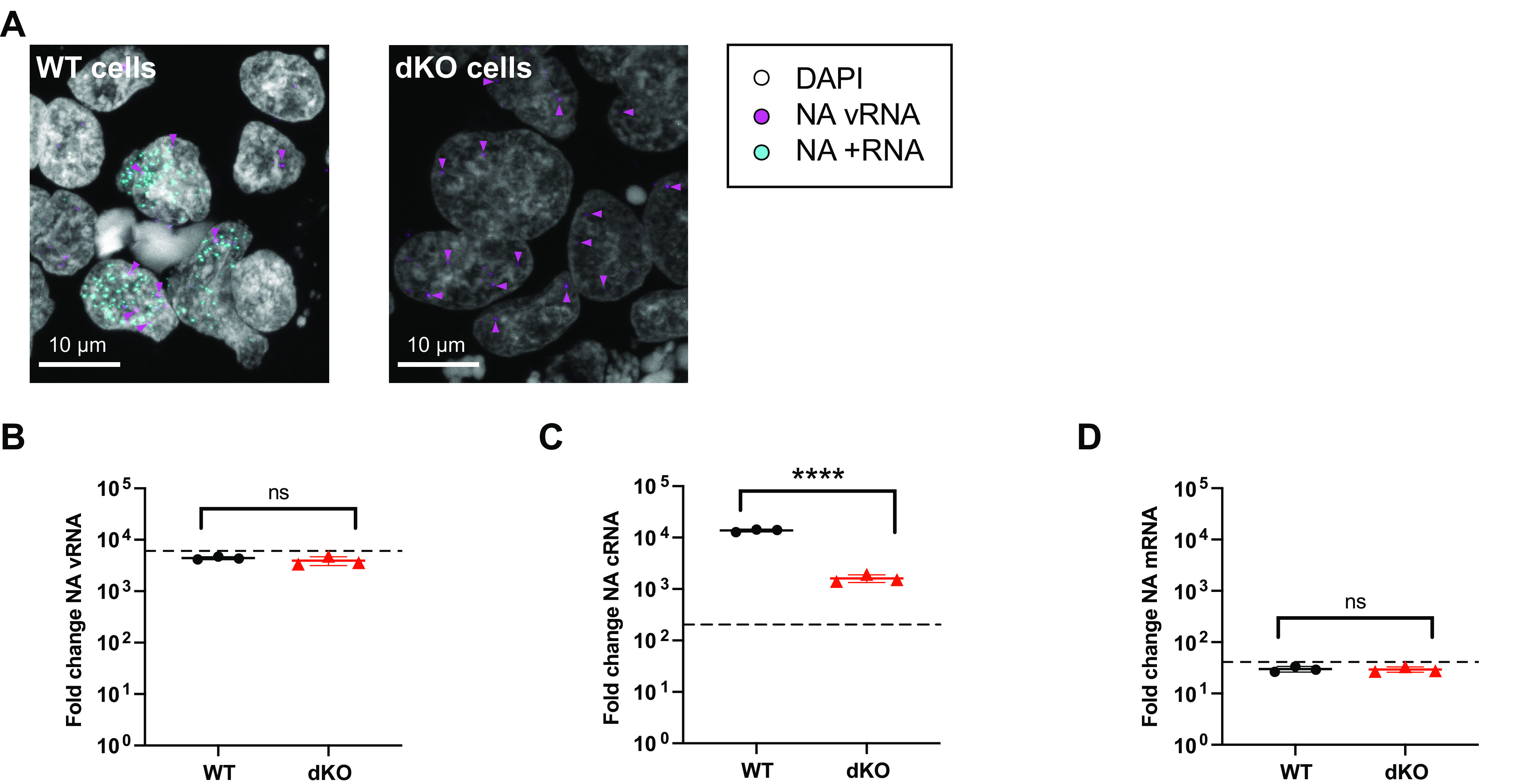
Human ANP32 proteins play a direct role in primary cRNA synthesis. ActD-treated cRNP stabilization assay in eHAP WT and dKO cells, following infection with PR8 (MOI = 3), 3 hpi. (A) Accumulation of segment 6 vRNA and +RNA in eHAP WT and dKO cells, analyzed using RNAscope. Magenta arrowheads highlight a subset of NA vRNA-stained puncta. Images are representative maximum-intensity projections. (B to D) Accumulation of segment 6 vRNA (B), cRNA (C), or mRNA (D) analyzed by RT-qPCR. The dotted line indicates the background RNA present in control samples transfected with a plasmid mix lacking PB1. The fold change was calculated versus mock-infected cells. *n* = 3 biological replicates, plotted as means ± the SD. Significance was assessed using an unpaired *t* test following log transformation. ns, not significant; ****, *P* < 0.0001.

To eliminate the possibility that ANP32 proteins are only required for vRNA synthesis, and the lack of cRNA accumulation in infected cells that lack ANP32 expression is an indirect effect, ActD-treated cRNP stabilization assays were performed in eHAP WT and dKO cells. +RNA accumulation was clearly detected 3 hpi in the nuclei of infected WT cells (Fig. 3A). In contrast, while incoming vRNPs were observed in the nuclei of dKO cells (magenta arrowheads), no +RNA staining was observed. When analyzed by RT-qPCR (Fig. 3B to D), a highly significant reduction in cRNA accumulation was observed in dKO cells compared to WT cells. As expected, no accumulation of NA vRNA or mRNA occurred over background levels (controls lacking transfected PB1, dotted line), in either cell type. Equal transfection efficiency was confirmed by Western blot (see Fig. S2). This corroborates that huANP32A/B are required for primary cRNA synthesis.

10.1128/mbio.03399-22.2 FIG S2 Matched Western blot for Fig. 3B to D. Download FIG S2, PDF file, 0.1 MB. Copyright © 2023 Swann et al. 2023 Swann et al. https://creativecommons.org/licenses/by/4.0/ This content is distributed under the terms of the Creative Commons Attribution 4.0 International license.

### ANP32 proteins are not required for primary transcription.

**FIG 4:**
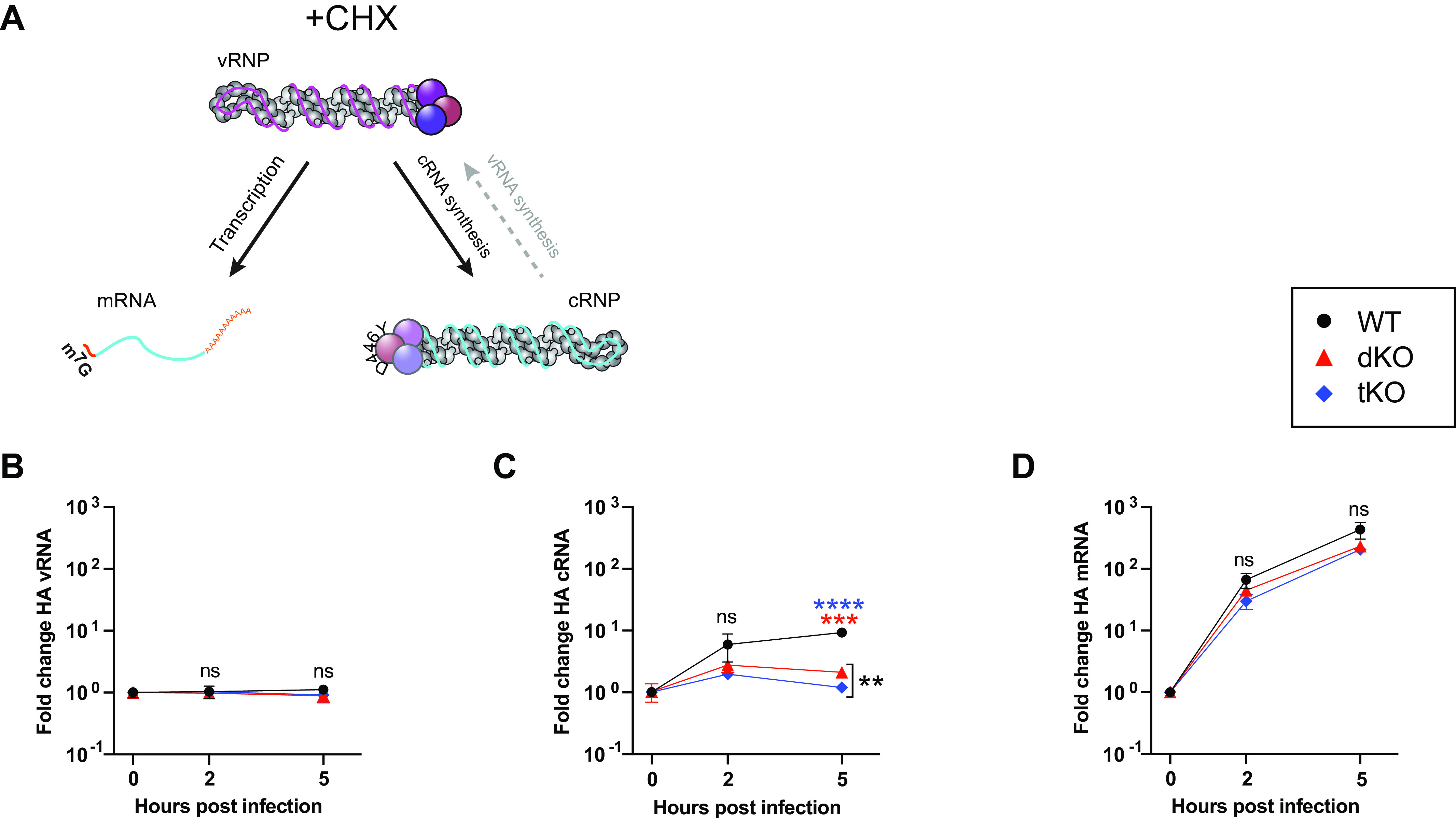
ANP32 proteins are not required for primary transcription. (A) Schematic illustrating influenza polymerase activity under the conditions of a cRNP stabilization assay with CHX. (B to D) cRNP stabilization assay with CHX in eHAP WT, dKO, and tKO cells. Segment 4 vRNA (B), cRNA (C), or mRNA (D) accumulation following infection with PR8 (MOI = 3) is shown. The fold change was calculated versus the input (0 hpi). *n* = 3 biological replicates, plotted as means ± the SD. Significant differences between cell types was assessed using multiple unpaired *t* tests following log transformation, corrected for multiple comparisons using the false discovery rate. ns, not significant; **, *P* < 0.01; ***, *P* < 0.001; ****, *P* < 0.0001.

Our infection data (Fig. 1) suggest that ANP32 proteins are not required for primary transcription. Nevertheless, at later infection time points, reduced mRNA accumulation is observed in dKO cells, compared to WT cells (Fig. 1C and F). To confirm this is an indirect effect, we chose to undertake a cycloheximide (CHX)-treated cRNP stabilization assay in eHAP wild type, dKO or in eHAP cells lacking expression of all three huANP32 proteins: A, B and E (eHAP tKO). Since CHX inhibits translation, in this version of the assay both the pioneering round of cRNA synthesis and primary transcription occur, but vRNA synthesis does not (Fig. 4A) (27). Accordingly, no accumulation of vRNA over input was observed in any of the cell types over time (Fig. 4B). In agreement with ActD-treated cRNP stabilization assays (Fig. 3C), a significant decrease in cRNA accumulation (>3-fold) was observed in cells that lack ANP32 expression compared to WT cells, by 5 hpi (Fig. 4C). In contrast, no significant difference was observed in mRNA accumulation in the presence or absence of ANP32 proteins from 0 to 5 hpi (Fig. 4D). Comparable transfection efficiency was confirmed by Western blot (see Fig. S3). Overall, these data corroborate a direct role for ANP32 proteins in cRNA synthesis using two different approaches (ActD and CHX inhibition assays) and two different methods (RT-qPCR and *in situ* FISH) and confirm that primary transcription does not require ANP32 proteins.

10.1128/mbio.03399-22.3 FIG S3 Matched Western blot for Fig. 4B to D. Download FIG S3, PDF file, 0.2 MB. Copyright © 2023 Swann et al. 2023 Swann et al. https://creativecommons.org/licenses/by/4.0/ This content is distributed under the terms of the Creative Commons Attribution 4.0 International license.

### Human ANP32 proteins play a direct role in primary cRNA synthesis in diverse IAV strains.

To broaden our findings to more biologically relevant strains of influenza virus, we quantified vRNA and cRNA accumulation in either eHAP WT or tKO cells following infection with a panel of reassortant viruses with PR8 external genes (to normalize entry) and internal genes representative of either: an H5N1 avian strain, A/turkey/England/50-92/1991(H5N1), engineered to carry PB2 627K for efficient replication in mammalian cells [H5N1(K)]; a pandemic human H1N1 strain, A/Eng/195/2009(H1N1) (H1N1[pdm09]); or a human H3N2 strain, A/Aichi/2/1968(H3N2) (see Fig. S4A to F). In the case of all three strains, a significant reduction in cRNA accumulation was already observed in tKO cells relative to WT by 2 hpi. In the case of the H5N1(K) and H1N1(pdm09) strains a very small upwards trend in cRNA accumulation was observed 2 to 5 hpi in the tKO cells. Therefore, to confirm the role for ANP32 proteins in supporting cRNA synthesis was direct, CHX-treated cRNP stabilization assays were performed (see Fig. S4G and H). In these assays, a highly significant reduction in cRNA accumulation was observed in the absence of ANP32 proteins, 5 hpi. Moreover, no accumulation of cRNA over background (dotted lines) was observed in the tKO cells. Overall, these data confirm that ANP32 proteins are required to directly support robust cRNA synthesis for a diverse range of IAV strains.

10.1128/mbio.03399-22.4 FIG S4 Human ANP32 proteins play a direct role in primary cRNA synthesis in diverse IAV strains. (A to F) Segment 6 vRNA accumulation (A, C, and E) or cRNA accumulation (B, D, and F) over time in eHAP WT versus tKO cells following infection with H5N1 (5092K) (A and B), H1N1 pdm09 (Eng195) (C and D), or H3N2 (Aichi68) (E and F) (MOI = 3). The fold change was calculated over input (0 hpi). *n* = 3 biological replicates, plotted as means ± the SD. Significance at 2 hpi was assessed using an unpaired *t* test following log transformation. (G) Segment 4 cRNA accumulation during a CHX-treated cRNP stabilization assay in eHAP WT and tKO cells, following infection with 5092K (MOI = 3). The fold change was calculated versus input (0 hpi). The dotted line indicates background levels of cRNA present in WT cells transfected with a control plasmid mix minus PB1, 5 hpi. *n* = 3 biological replicates, plotted as means ± the SD. Significance was assessed using an unpaired *t* test following log transformation. (H) Segment 6 cRNA accumulation during a CHX-treated cRNP stabilization assay in eHAP WT and tKO cells, following infection with Eng195 (MOI = 3). The fold change was calculated versus input (0 hpi). Black dotted line indicates background levels of cRNA present in WT cells transfected with a control plasmid mix minus PB1, 5 hpi; green dotted line indicates background levels of cRNA present in WT cells transfected with a control plasmid mix minus NP, 5 hpi. *n* = 3 biological replicates, plotted as means ± the SD. Significance was assessed using an unpaired *t* test following log transformation. *, *P* < 0.05; ***, *P* < 0.001; ****, *P* < 0.0001. Download FIG S4, PDF file, 0.1 MB. Copyright © 2023 Swann et al. 2023 Swann et al. https://creativecommons.org/licenses/by/4.0/ This content is distributed under the terms of the Creative Commons Attribution 4.0 International license.

### Avian polymerase is not restricted in cRNA synthesis in mammalian cells.

**FIG 5:**
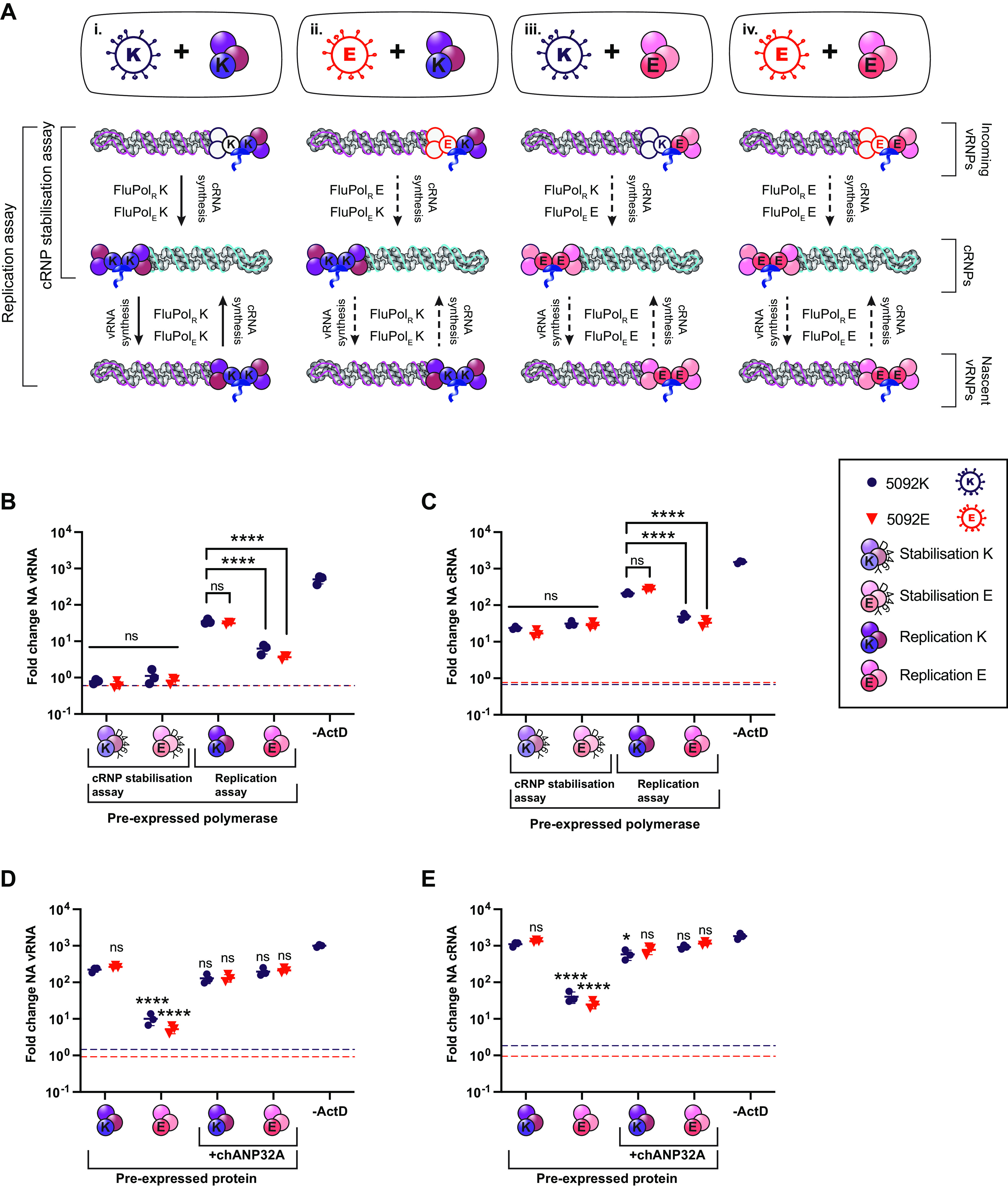
Avian polymerase is not restricted in cRNA synthesis in mammalian cells. (A) Schematic outlining assay setup and expected polymerase combinations during replication. As indicated on the left, in the cRNP stabilization assay, only the first layer of activity (primary cRNA synthesis) will occur. Both layers of activity can occur in replication assays. Unknown polymerase activity is indicated by a dashed black arrow. (B and C) Simultaneous cRNP stabilization and replication assays with ActD, 6 hpi. Segment 6 vRNA (B) and cRNA (C) accumulation following infection with either 5092E or 5092K as indicated (MOI = 0.1) is shown. The dotted line indicates levels of vRNA/cRNA present in a control lacking PB2 in the transfection mix. The fold change was calculated versus input (0 hpi). *n* = 3 biological replicates, plotted as means ± the SD. Significance was assessed using one-way ANOVA with Dunnett’s multiple-comparison test, following log transformation. (D and E) Replication assays with ActD and chANP32A, 6 hpi. Segment 6 vRNA (D) and cRNA (E) accumulation following infection with either 5092E or 5092K as indicated (MOI = 0.1) is shown. The dotted line indicates the levels of vRNA/cRNA present in a control lacking PB2 in the transfection mix. The fold change was calculated over input (0 hpi). *n* = 3 biological replicates, plotted as means ± the SD. Significance was assessed using one-way ANOVA with Dunnett’s multiple-comparison test, following log transformation. Pre-expressed polymerase mixes: stabilization K = PB2 627K, PB1 D446Y, PA, and NP; stabilization E = PB2 627E, PB1 D446Y, PA, and NP; replication K = PB2 627K, PB1, PA, and NP; replication E = PB2 627E, PB1, PA, and NP. ns, not significant; *, *P* < 0.05; ****, *P* < 0.0001.

Restriction of avian signature FluPol (FluPol 627E) in mammalian cells is attributed to incompatibility with mammalian ANP32 orthologues. Previous work has mapped restriction specifically to the level of vRNA synthesis (21, 22, 28). However, as we have confirmed that ANP32 is required to support both cRNA and vRNA synthesis, we would expect FluPol 627E to be restricted in both steps of replication. To investigate this apparent contradiction, we used a pair of isogenic viruses based on the avian strain A/turkey/England/50-92/1991(H5N1) (5092) that differ only in the residue at PB2 position 627: either the wild-type PB2 627E (here referred to as 5092E) or the humanizing mutation PB2 E627K (5092K) that adapts FluPol for efficient support by human ANP32 proteins (16, 29). We undertook simultaneous cRNP stabilization and replication assays in human 293T cells (where only huANP32A/B/E proteins, that are incompatible with FluPol 627E, are available). In these assays, during primary cRNA synthesis, FluPol_E_ is provided by the pre-expressed polymerase while FluPol_R_ is brought in on the vRNPs from the infecting virus. Thus, by mixing and matching the input virus PB2 627 identity with that of the pre-expressed polymerase, heterologous combinations of FluPol can be achieved (depicted in the first line of Fig. 5A, “incoming vRNPs”). Thus, the effect of the 627 residue of either FluPol_R_ or FluPol_E_ on the pioneering round of cRNA synthesis can be differentiated. In subsequent rounds of replication (that only occur in the replication assay), both FluPol signatures are dictated by the genotype of the pre-expressed polymerase (Fig. 5A, bottom two lines).

In Fig. 5B and C, cRNP stabilization assays are reported on the left-hand side of the graph, while replication assays are reported on the right-hand side of the graph. The cRNP stabilization assay measures the pioneering round of cRNA synthesis, during which FluPol_E_ is provided by the pre-expressed polymerase while FluPol_R_ is brought in on the vRNPs from the infecting virus (Fig. 5A, “cRNP stabilization assay”). No vRNA accumulated in this assay, as expected (Fig. 5B). Interestingly, neither the PB2 signature of FluPol_R_ nor FluPol_E_ impacted cRNP stabilization, with all four FluPol combinations accumulating equivalent amounts of cRNA (Fig. 5C). Even the fully avian combination of 5092E virus with pre-expressed FluPol 627E (Fig. 5Aiv) accumulated equivalent amounts of cRNA as the fully humanized combination of 5092K virus plus FluPol 627K (Fig. 5Ai). This demonstrates that the residue at position 627 on PB2 does not impact cRNA synthesis in human cells, i.e., avian FluPol is fully compatible with huANP32 proteins for cRNA synthesis and stabilization.

In the replication assay, samples with pre-expressed FluPol 627K accumulated equivalent quantities of vRNA and cRNA, regardless of whether the incoming virus was 5092K or 5092E (Fig. 5Ai and ii, B, and C). Similarly, samples with pre-expressed FluPol 627E were equally impaired in vRNA and cRNA synthesis, independent of infecting virus signature (Fig. 5Aiii and iv, B, and C). It has previously been described that host restricted FluPol is impaired in nuclear import, due to incompatibilities with the importin-alpha isoforms present in human cells (30). However, this cannot explain the data obtained here, since FluPol 627E was fully functional in supporting the pioneering round of cRNA synthesis within the nucleus during cRNP stabilization assays. Since replication assays allow multiple cycles of replication, the reduced cRNA synthesis observed with pre-expressed FluPol 627E is likely due to the secondary effect of reduced vRNP template. In further replication assays, exogenous expression of chANP32A was able to rescue replication for all FluPol combinations, confirming that restriction maps to ANP32 (Fig. 5D and E).

## DISCUSSION

Here, we have used cell lines that lack expression of ANP32 proteins to unambiguously confirm huANP32 is required to support cRNA synthesis, the first step of replication, by influenza virus polymerase. Nevertheless, we did not observe restriction of avian virus cRNA synthesis in human cells, suggesting that host range restriction imparted by species differences in ANP32 acts only at the level of vRNA synthesis.

In the structure of the chANP32A-FluPol complex described by Carrique et al. (11), the ANP32 LRR domain bridges an asymmetric dimer of two FluPol heterotrimers. This structure has been interpreted as a “replicase” complex that coordinates encapsidation of nascent cRNPs and vRNPs. Our results demonstrating that ANP32 is indeed required for cRNA synthesis is consistent with such an interpretation. In the cryo-electron microscopy structure, the unstructured, flexible low-complexity acidic region (LCAR) domain of chANP32A is largely unresolved, although there is additional density present in a groove formed between the two FluPol 627 domains. Since the groove is negatively charged in FluPol 627E, the authors suggest the acidic huANP32 LCAR is likely incompatible with this interaction, whereas this clash is averted by the 33-amino-acid insertion in avian chANP32A (11). Such a model for host restriction is also supported by nuclear magnetic resonance analysis (31). Our data suggest that, unexpectedly, the step of cRNA synthesis is tolerant of the charge mismatches between the huANP32 LCAR and FluPol 627E. This conclusion aligns with the observation that exogenous expression of chANP32A in human cells does not further stimulate FluPol 627E cRNA synthesis (28). Thus, we propose the optimized 627-LCAR interaction could be required for a distinct mechanistic role that is specific to vRNA synthesis.

Previous studies have suggested host restricted avian FluPol can generate cRNA in human cells but that the resulting cRNPs are aberrant (21, 23, 28). Our RT-qPCR approach relies on primers that bind to the very 3′ end of the cRNA; thus, any signal is suggestive of a full-length cRNA segment, although we cannot exclude the presence of internal deletions upstream of the ~100-bp qPCR amplified fragment. We also cannot exclude that a full-length cRNA is produced but incorrectly assembled and therefore rendered nonfunctional. Nonetheless, our data demonstrate that cRNA produced from 5092E is functional for onwards replication so long as the encapsidating FluPol bears the mammalian-adapted 627K signature (i.e., the scenario depicted in Fig. 5Aii). We suggest that cRNPs produced from FluPol_R_ 627E in mammalian cells are therefore fully functional. Alternatively, it is possible that the presence of pre-expressed FluPol 627K in this scenario is sufficient to overcome host restriction, either because the 627 signature of FluPol_E_ dictates host restriction or a single PB2 627K residue present in either FluPol_R_ or FluPol_E_ is sufficient to negate the charge clash. We note that in cRNP stabilization assays equivalent quantities of cRNA accumulate in mammalian cells regardless of whether FluPol_R_, FluPol_E_, or both FluPol molecules in the replication complex bear the avian-like 627E signature, but no cRNA accumulation occurs in the complete absence of ANP32 proteins. This suggests there is at least some functional interaction between huANP32 proteins and FluPol 627E.

**FIG 6:**
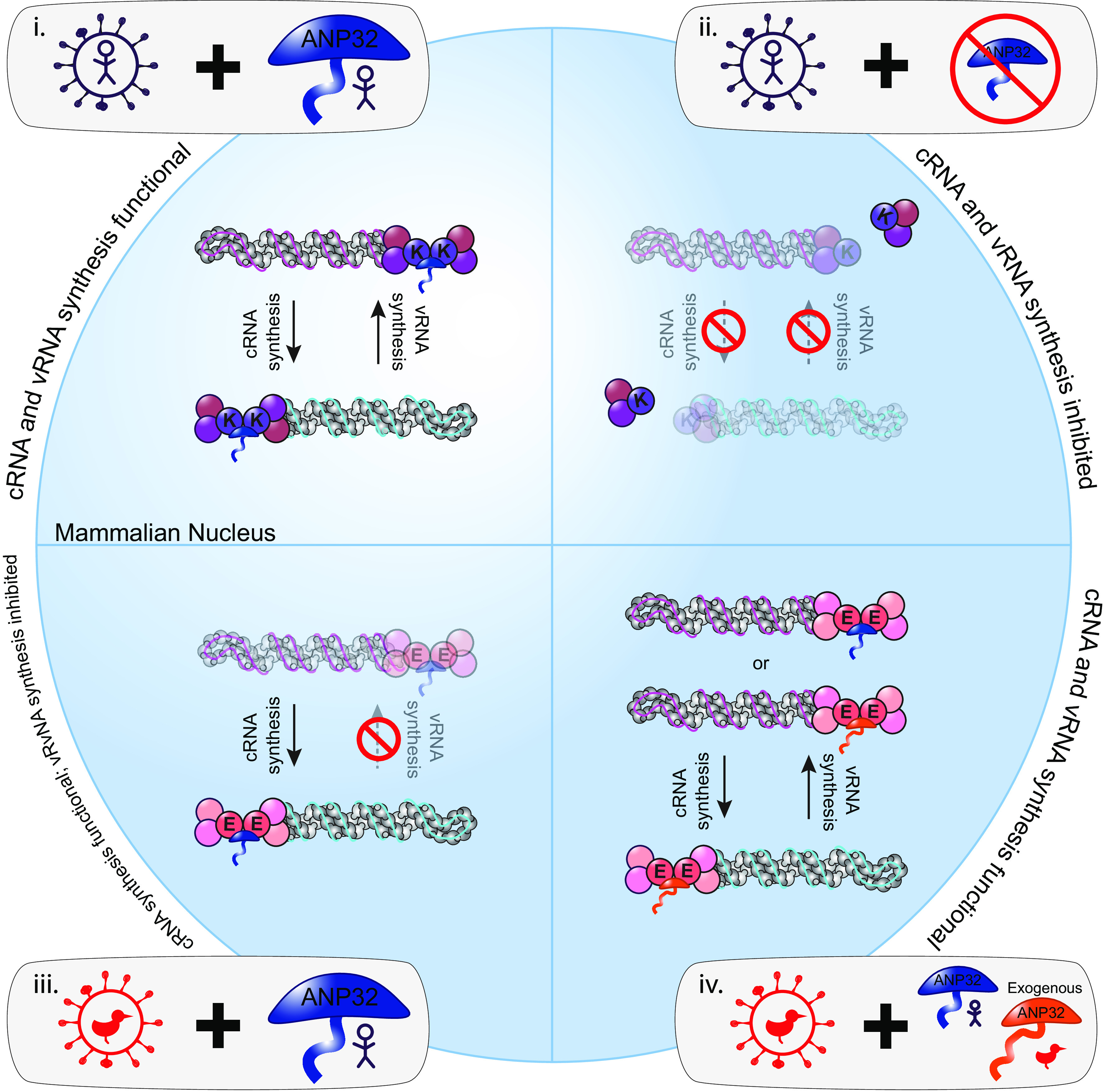
Summarizing schematic. (i) When human-adapted influenza viruses encoding a lysine at position 627 of the PB2 polymerase protein (PB2 627K) infect wild-type human cells in which only human ANP32 proteins are present, both cRNA and vRNA synthesis can occur. (ii) When influenza viruses (PB2 627K) infect human cells lacking ANP32 proteins, neither cRNA nor vRNA accumulate, demonstrating that ANP32 is required to support both cRNA and vRNA synthesis. (iii) When avian influenza viruses encoding a glutamic acid at position 627 of the PB2 polymerase protein (PB2 627E) infect wild-type human cells in which only human ANP32 proteins are present, cRNA synthesis but not vRNA synthesis can occur. Since scenario ii illustrates that ANP32 proteins are required for cRNA synthesis, this suggests that avian polymerase can functionally interact with human ANP32 proteins to support cRNA synthesis. However, avian polymerase cannot utilize human ANP32 proteins to support vRNA synthesis; thus, host restriction is imposed on this step of replication. (iv) In the presence of exogenously expressed avian ANP32A, avian influenza viruses (PB2 627E) can undergo both cRNA and vRNA synthesis, confirming restricted vRNA synthesis in scenario iii maps to aberrant interactions with human ANP32 proteins.

In summary, this work has established that huANP32 is required for cRNA synthesis, as well as vRNA synthesis, in human cells and that huANP32 paralogues are sufficient for supporting stabilization of cRNA produced by an avian influenza FluPol (Fig. 6). This suggests host restriction of avian FluPol 627E acts specifically at the level of vRNA synthesis. We have also established an RNA FISH assay which allows visualization of FluPol replication products. This allows single-cell, spatial information to be collated, which in the future could be used to improve our understanding of the spatial elements of FluPol regulation.

## MATERIALS AND METHODS

### Cells and cell culture.

Human-engineered haploid cells (eHAP cells; Horizon Discovery), eHAP cells with both huANP32A and huANP32B (eHAP dKO) ablated via CRISPR-Cas9, as previously described (14), or eHAP cells with huANP32A, huANP32B, and huANP32E ablated via CRISPR-Cas9 (eHAP tKO) (gift from Ecco Staller and Ervin Fodor) were maintained in Iscove modified Dulbecco medium (Thermo Fisher) supplemented with 10% fetal bovine serum (FBS; Labtech), 1% penicillin-streptomycin (pen-strep; Gibco), and 1% nonessential amino acids (NEAA; Gibco). Human embryonic kidney (293Ts; ATCC) and Madin-Darby canine kidney (ATCC) cells were maintained in Dulbecco modified Eagle medium supplemented with 10% FBS, 1% pen-strep, and 1% NEAA. When used for infection, 293T cells were cultured on poly-l-lysine-coated plates to aid adherence. All cells were maintained at 37°C and 5% CO_2_.

### Plasmids.

pCAGGS expression plasmids encoding the polymerase subunits (PB2, PB1, and PA) and NP from PR8 were subcloned from pPolI plasmids. pCAGGS expression plasmids encoding 50-92 and Eng195 PB2, PB1, PA, and NP have previously been described (32, 33). The catalytic mutant PB1 D446Y has been described (34, 35). pCAGGS expression plasmid encoding FLAG-tagged chicken ANP32 has also been described (16).

### Viral infections.

The full strain names of viruses used in this study are A/Puerto Rico/8/1934(H1N1) (PR8), A/turkey/England/50-92/1991(H5N1) (50-92), A/Aichi/2/1968(H3N2) (Aichi68), and A/Eng/195/2009(H1N1) (Eng195). For experiments using 50-92, all infections were performed with recombinant viruses containing the HA, NA, and M segments from PR8, the PB1, PA, NP, and NS segments from 50-92 and either the WT 50-92 PB2 segment containing a lysine at position 627 (5092E) or a modified PB2 with a glutamic acid at position 627 (5092K), as previously described (29). For experiments using Aichi68 and Eng195, all infections were performed with recombinant viruses containing the HA and NA segments from PR8, and Aichi68 or Eng195 internal genes, respectively. For infections, virus was diluted in serum-free media to the correct multiplicity of infection (MOI; described in the figure legends). For comparative 5092E/K experiments, viral inputs were normalized based on genome copy number. To synchronize infection, viral inoculation was performed at 4°C. In brief, cells were preincubated at 4°C for 15 min before the addition of the viral inoculum and a further incubation at 4°C for 45 min. Viral inoculum was then replaced with prewarmed full media, and infected plates were incubated at 37°C and 5% CO_2_. At the appropriate time point, cells were processed for RT-qPCR analysis, imaging analysis, or immunoblotting as described below.

### Replication/cRNP stabilization assays.

For replication/cRNP stabilization assays, cells were transfected using Lipofectamine 3000 (Invitrogen) with pCAGGS expression plasmid mixtures encoding polymerase components in the ratios 2:2:1:4, PB2:PB1:PA:NP, where 1 = 20, 40, or 80 ng (24-well plate, 12-well plate, or 6-well plate, respectively). For experiments including chANP32A, pCAGGS expression plasmid encoding FLAG-tagged chANP32A was included in the transfection mix at a ratio of 4. For replication assays, WT PB1 plasmid was pre-expressed, while for cRNP stabilization assays catalytically dead polymerase (PB1 D446Y) was pre-expressed. For experiments using eHAP cells, 20 h posttransfection cells were preincubated in medium containing actinomycin D (10 μg/mL), cycloheximide (100 μg/mL) or dimethyl sulfoxide control as indicated. One hour later, cells were infected as described above, with the addition of the appropriate concentration drug in the inoculum and subsequent prewarmed media. For experiments using 293T cells, preincubations were omitted, and actinomycin D was used at 5 μg/mL. At the appropriate time point, cells were processed for RT-qPCR analysis, imaging analysis, or immunoblotting as described below.

### Tagged RT-qPCR against vRNA, cRNA, and mRNA.

For RT-qPCR analysis, 293T or eHAP cells were cultured in 24-well plates, with each condition in triplicate. After infection/transfection, cells were lysed using buffer RLT or RLT Plus (Qiagen) and frozen at −80, and then the total RNA was extracted using either an RNeasy RNA extraction kit (Qiagen) with 30 min on-column DNase I digestion or a QIAsymphony RNA kit (Qiagen). Quantification for segment 4 and segment 6 vRNA, cRNA, and mRNA was based on the tagged primer approach developed by Kawakami et al. (25). For each sample, four reverse transcription reactions were set up using 200 ng RNA/reaction, RevertAid H Minus Reverse Transcriptase (Thermo Scientific) (used according to the manufacturer’s instructions), plus a tagged primer targeting either vRNA or cRNA, a tagged poly(dT) (for viral mRNA) or an untagged poly(dT) (for the GAPDH internal control). For NA vRNA, cRNA, and mRNA, the primers used were GGCCGTCATGGTGGCGAATGAAACCATAAAAAGTTGGAGGAAG, GCTAGCTTCAGCTAGGCATCAGTAGAAACAAGGAGTTT, and CCAGATCGTTCGAGTCGTTTTTTTTTTTTTTTTTT, respectively (tags underlined), while the primers used for HA vRNA, cRNA, and mRNA were (GGCCGTCATGGTGGCGAATGGAGAGTGCCCAAAATACGT, GCTAGCTTCAGCTAGGCATCAGTAGAAACAAGGGTGTT, and CCAGATCGTTCGAGTCGTTTTTTTTTTTTTTTTTT, respectively). Tagged cDNA was then diluted 1 in 10 and quantified using real-time qPCR using Fast SYBR green master mix (Thermo Scientific). The primer pairs used were CCTTCCCCTTTTCGATCTTG/GGCCGTCATGGTGGCGAAT (NA vRNA), CTTTTTGTGGCGTGAATAGTG/GCTAGCTTCAGCTAGGCATC (NA cRNA), CTTTTTGTGGCGTGAATAGTG/CCAGATCGTTCGAGTCGT (NA mRNA), CATACCATCCATCTATCATTCC/GGCCGTCATGGTGGCGAAT (HA vRNA), GGGGGCAATCAGTTTCTG/GCTAGCTTCAGCTAGGCATC (HA cRNA), GATTCTGGCGATCTACTCAACTGTC/CCAGATCGTTCGAGTCG (HA mRNA), and AATCCCATCACCATCTTCCA/TGGACTCCACGACGTACTCA (GAPDH). qPCR analysis was carried out in duplicate or triplicate on a Viia 7 real-time PCR system (Thermo Fisher). Fold changes in gene expression relative to either input (0 hpi) or mock-infected controls (as indicated in the figure legends) were calculated using the 2^–ΔΔ^*^CT^* method with GAPDH expression as internal control.

### RNAscope/immunofluorescence costaining.

For imaging analysis, cells were cultured on glass coverslips coated in poly-l-lysine in 12-well plates. At the appropriate time point, infected cells were washed in phosphate-buffered saline (PBS; Gibco) and fixed in 4% paraformaldehyde for 30 min, prior to further washes in PBS and dehydration in an ethanol gradient (50% ethanol [EtOH], 5 min, 70% EtOH, 5 min, 100% EtOH, 5 min, fresh 100% EtOH, 10 min). Cover slips were stored in 100% ethanol at −20°C until further processing. For RNAscope/immunofluorescence costaining, RNA was stained first using RNAscope probes (ACDBio). Probes were designed to target PR8 NA vRNA (channel 1) and PR8 NA cRNA/mRNA (+RNA) (channel 2). Cover slips were rehydrated in an ethanol gradient (70% EtOH, 2 min, 50% EtOH, 2 min, PBS, 10 min), treated with protease III diluted 1 in 15 in PBS and staining undertaken using the fluorescent multiplex kit v1, following the manufacturer’s instructions up until and including incubation in the final fluorophore mixture (Fl-Amp4). At this point, coverslips were blocked in PBS with 2% bovine serum albumin and 0.1% Tween for 30 min, incubated in rabbit α-PB2 (catalog no. GTX125926; Genetex) antibody for 1 h at room temperature, followed by goat α-rabbit AF647 (Invitrogen) plus DAPI (4′,6′-diamidino-2-phenylindole) for 1 h at room temperature. Images were obtained using a Leica SP5 inverted confocal microscope and processing undertaken using FIJI software (36, 37).

### Immunoblot analysis.

To confirm equivalent protein expression during replication/cRNP stabilization assays, cells transfected in parallel were lysed with homemade radioimmunoprecipitation assay buffer (150 mM NaCl, 1% NP-40, 0.5% sodium deoxycholate, 0.1% sodium dodecyl sulfate, 50 mM Tris [pH 7.4]) supplemented with an EDTA-free protease inhibitor cocktail tablet (Roche). Lysates were clarified and then mixed with 4× Laemmli sample buffer (Bio-Rad) with 10% β-mercaptoethanol. Membranes were probed with rabbit α-vinculin (EPR8185; Abcam) or mouse α-tubulin (ab7291; Abcam) and mouse α-NP (C43, Abcam), followed by near-infrared secondary antibodies (IRDye 680RD goat anti-rabbit [IgG] secondary antibody [Abcam] and IRDye 800CW goat anti-mouse [IgG] secondary antibody [Abcam]). Due to similarities in size, blots probed for NP and tubulin were run in duplicate and probed on separate membranes. Blots were visualized using an Odyssey imaging system (Li-Cor Biosciences).

### Statistical analysis.

Statistics throughout this study were assessed using one-way analysis of variance (ANOVA) or Student *t* test as described in the figure legends.
