# Supplementary figures and images for: Avian Influenza A Virus Polymerase Can Utilize Human ANP32 Proteins To Support cRNA but Not vRNA Synthesis
Source: mBio. 2023 Jan 16;14(1):e03399-22. doi: 10.1128/mbio.03399-22 (PMC9973007; doi:10.1128/mbio.03399-22)

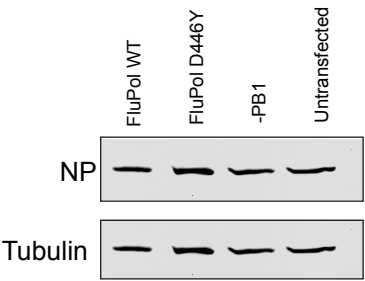

Supplement: FIG S1 [file mbio.03399-22-s0001.pdf]

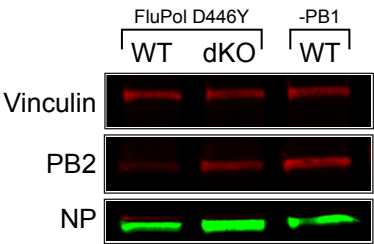

Supplement: FIG S2 [file mbio.03399-22-s0002.pdf]

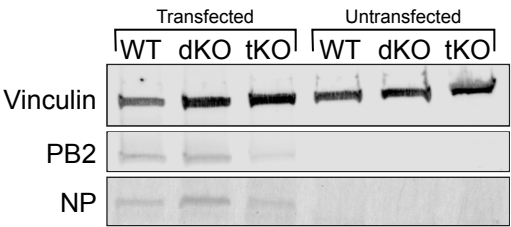

Supplement: FIG S3 [file mbio.03399-22-s0003.pdf]

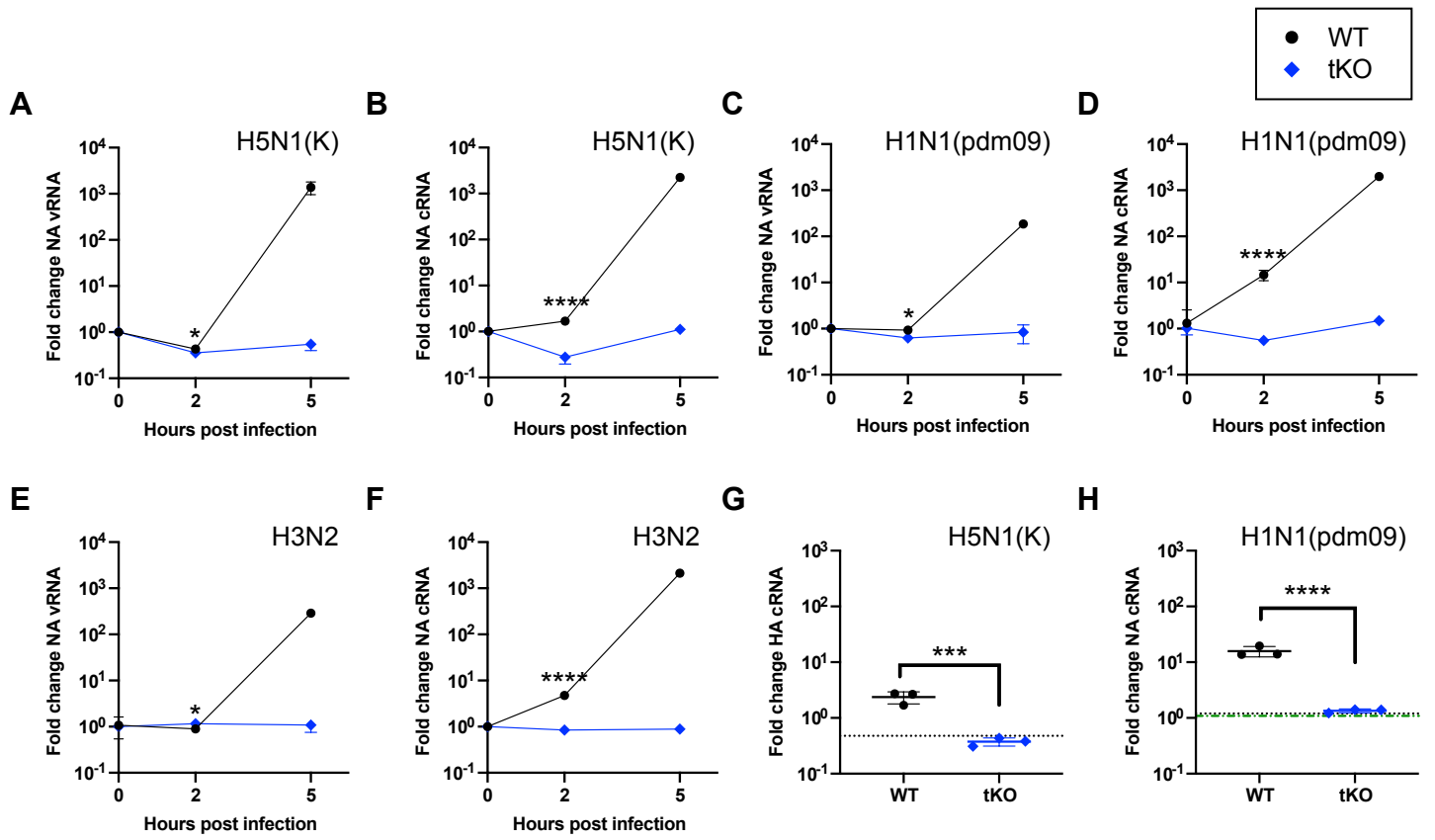

Supplement: FIG S4 [file mbio.03399-22-s0004.pdf]
